# Characterization of the PRMT Gene Family in Rice Reveals Conservation of Arginine Methylation

**DOI:** 10.1371/journal.pone.0022664

**Published:** 2011-08-11

**Authors:** Ayaz Ahmad, Yuzhu Dong, Xiaofeng Cao

**Affiliations:** 1 State Key Laboratory of Plant Genomics and National Center for Plant Gene Research, Institute of Genetics and Developmental Biology, Chinese Academy of Sciences, Beijing, China; 2 Graduate University of the Chinese Academy of Sciences, Beijing, China; Duke University, United States of America

## Abstract

Post-translational methylation of arginine residues profoundly affects the structure and functions of protein and, hence, implicated in a myriad of essential cellular processes such as signal transduction, mRNA splicing and transcriptional regulation. Protein arginine methyltransferases (PRMTs), the enzymes catalyzing arginine methylation have been extensively studied in animals, yeast and, to some extent, in model plant *Arabidopsis thaliana*. Eight genes coding for the PRMTs were identified in *Oryza sativa*, previously. Here, we report that these genes show distinct expression patterns in various parts of the plant. *In vivo* targeting experiment demonstrated that GFP-tagged OsPRMT1, OsPRMT5 and OsPRMT10 were localized to both the cytoplasm and nucleus, whereas OsPRMT6a and OsPRMT6b were predominantly localized to the nucleus. OsPRMT1, OsPRMT4, OsPRMT5, OsPRMT6a, OsPRMT6b and OsPRMT10 exhibited *in vitro* arginine methyltransferase activity against myelin basic protein, glycine-arginine-rich domain of fibrillarin and calf thymus core histones. Furthermore, they depicted specificities for the arginine residues in histones H3 and H4 and were classified into type I and Type II PRMTs, based on the formation of type of dimethylarginine in the substrate proteins. The two homologs of OsPRMT6 showed direct interaction *in vitro* and further titrating different amounts of these proteins in the methyltransferase assay revealed that OsPRMT6a inhibits the methyltransferase activity of OsPRMT6b, probably, by the formation of heterodimer. The identification and characterization of PRMTs in rice suggests the conservation of arginine methylation in monocots and hold promise for gaining further insight into regulation of plant development.

## Introduction

Post-translational methylation at arginine significantly influences the structure and functions of affected protein, by changing the bulkiness and hydrophobicity of modified residues, and hence modulates a myriad of essential biological processes, including transcriptional regulation, RNA metabolism, DNA repair, signal transduction, protein sorting, and apoptosis *etc* (reviewed in [1]–[6]). During this reaction methyl group is transferred from S-adenosyl-L-methionine (SAM), a universal methyl donor, to the arginines in the substrate proteins. The family of enzymes catalyzing the methylation at arginine residues is called protein arginine methyltransferases (PRMTs). Protein arginine methylation was reported in 1966, but, the first gene coding for the arginine methyltransferase enzyme was discovered only in 1996 (reviewed in [7]). Following their discovery, emphasis has been given to characterize PRMTs in different organisms, in order to understand their important biological functions. Hitherto, 11 PRMT members, differing in sequences and substrate specificities, have been characterized in humans [1]; however, the molecular mechanisms through which they regulate cellular processes are still largely unknown.

Arginine residue contains 3 nitrogen atoms which can replace their 5 hydrogen atoms and form different methylated arginines [3]. Based on the formation of different methylarginines, protein arginine methyltransferases can be divided into four types [8]. Type I PRMTs, including PRMT1, PRMT2, PRMT3, PRMT4/coactivator-associated arginine methyltransferase 1 (CARM1), PRMT6, PRMT8 and RMT1, catalyze the formation of ω-*N^G^*-monomethyl arginine (MMA) and asymmetric ω-*N^G^*, *N^G^*-dimethylarginine (aDMA). Type II PRMTs result in the formation of monomethyl arginine and symmetric ω-*N^G^*, *N^′G^*-dimethylarginine (sDMA), this class includes PRMT5 and *Saccharomyces cerevisiae* histone synthetic lethal 7 (Hsl7) [9]. Type III PRMTs form only ω-*N^G^*-monomethyl arginine; *Trypanosoma brucie* homolog of human PRMT7, TbPRMT7 displays this type of activity [10]. Type IV enzymes catalyze the formation of δ-*N^G^*-monomethyl arginine and PRMT2 (RMT2) from *Saccharomyces cerevisiae* and *Candida albicans* has been found to harbor this activity so far [11], [12].

PRMTs methylate a large number of essential proteins, most of them are either RNA binding proteins or involved in transcription [4], [5], [13]. PRMT1 is the main arginine methyltransferase, responsible for at least 85% of all the PRMT activity in mouse [14], which catalyses the transfer of methyl group to a wide variety of substrates, including histones as well as non-histone proteins [2]–[4], [15], [16]). PRMT1 plays a critical role in early mouse development since the growth of *PRMT1*-null embryo was arrested shortly after implantation [14]. Human PRMT2 methylates histone H4 [17] and heterogeneous nuclear ribonucleoprotein E1B-AP5 [18] and acts as a co-activator of the androgen and estrogen receptors [19], [20]. PRMT3 catalyzes the methylation of ribosomal protein S2 (rpS2) [21], inhibits its ubiquitination [22] and regulates ribosome biosynthesis at a stage beyond pre-rRNA processing [23]. PRMT4/CARM1 regulates many cellular processes (reviewed in [2], [3]) including gene expression and pre-mRNA splicing [24], [25] and *PRMT4*/*CARM1*-null mice were shown to be deficient in some aspects of estrogen-responsive gene expression [26]. PRMT5 plays an important role in spliceosome assembly by methylating sphingomyelin (Sm) and survival of motor neuron (SMN) [27]. It regulates interleukin 2 (IL-2) gene expression [28] and is required for myogenesis because it facilitates ATP-dependent chromatin remodeling [29]. PRMT6 mediated methylation plays role in defense against HIV1 by regulating the HIV1 gene expression [30]. PRMT7 methylates histones, myelin basic protein, Glycine-Arginine-Rich domain (GAR), and spliceosomal protein SmB. PRMT7 was reported to probably playing role in male germ line imprinted gene methylation [31]. PRMT8 remains associated with membranes due to myristoylation and has an adopter role for nuclear proteins at the cell membrane in addition to its methyltransferase activity [32]. The protein encoded by the genes 4q31and 9p13.2 have been named as human PRMT9 and PRMT10, however, they remain to be characterized.

 Since they regulate many important biological processes in animals and yeast, great prominence has been given to explore the significance of PRMTs in model plant *Arabidopsis thaliana*, recently. *Arabidopsis thaliana* genome contains two homologs of human *PRMT1*; a major type I PRMT, *AtPRMT1a* and *AtPRMT1b*. AtPRMT1a and AtPRMT1b methylate the RNA methyltransferase, fibrillarin and histone H4 at R3 *in vitro* [33]. Moreover, AtPRMT1b (PRMT11) interacts with methyl-DNA-binding protein 7 (MBD7) [34], however, neither single nor double mutant of *AtPRMT1a* and *AtPRMT1b* show any visible phenotype under long day conditions (unpublished data, Yong Zhang and Xiaofeng Cao). AtPRMT4a and AtPRMT4b are the Arabidopsis homologs of human PRMT4/CARM1, which methylate histone H3 at R2, R17 and R26 *in vitro* and are required for methylation at H3R17 *in vivo*. Their double mutant shows an *FLC*-dependent late flowering phenotype [35]. AtPRMT5/(Shk1binding protein 1) Skb1 is a type II PRMT, resulting in the formation of symmetric dimethylarginines in the substrate proteins; and its knockout mutants exhibit pleiotropic phenotype including retarded growth, dark green and curly leaves and *FLC*-dependent late flowering [36], [37]. Lines of evidences show that arginine methylation mediated by PRMT5 is required for normal pre-mRNA splicing both in plants and animals [38], [39] and that the late flowering phenotype of *atprmt5-1* and *atprmt5-2* might be due to the splicing defects in the RNA processing-related flowering time regulators [38]. It has also been shown that the *FLC* expression is up-regulated by the splicing defects in *FLK* in the *atprmt5* mutant. It was reported recently that PRMT5 is a critical determinant of circadian period in Arabidopsis and Drosophila [39], [40] and it probably links the circadian cycle to the alternative splicing [39]. Furthermore, PRMT5/Skb1 regulates transcription and pre-mRNA splicing by symmetrically dimethylating histone H4R3 and small nuclear ribonucleoprotein LSM4 and confers high salt tolerance in Arabidopsis [41]. Another plant-specific type I PRMT, AtPRMT10 was shown to asymmetrically dimethylate histone H4 at R3 and regulate the Arabidopsis flowering time in an *FLC*-dependent manner [42].

*Oryza sativa* (rice), a representative of the monocots, is one of the most important cereals, feeding more than half of the world population and has the smallest genome in the cultivated cereals. In this study, we aligned the amino acid sequences of eight *OsPRMT* genes and found that they contain the conserved PRMT signature motifs. We compared the expression patterns, subcellular localization, *in vitro* enzymatic activity, and specificity of the *Oryza sativa* PRMT homologs for the arginine residues in histones H3 and H4. Our study suggests that, like animals and Arabidopsis, this conserved family of PRMTs may play diverse roles in transcriptional regulation and other vital cellular processes by methylating histones and non-histone proteins *in vivo*; studying the knockdown and knockout mutants will further strengthen this notion.

## Materials and Methods

### Amino acid sequence alignment and chromosomal distribution of the OsPRMTs

The amino acid sequences of the eight putative *OsPRMT* genes reported earlier were aligned in the multiple sequences alignment tool CLUSTALX1.83, with default parameters. The conserved PRMTs signature motifs were identified and edited manually with GeneDoc software.For finding the percent identity/similarity to the Arabidopsis PRMTs, the amino acid sequences of OsPRMTs were aligned in BLAST (bl2seq) at the NCBI website. Chromosomal distribution of these genes was determined by making use of the NCBI mapviewer tool on the NCBI website http://www.ncbi.nlm.nih.gov/projects/mapview/map_search.cgi?taxid=4530.

### RNA extraction, cDNA synthesis and relative gene expression patterns analysis

Total RNAs were isolated from different tissues, namely root, shoot, young leaf, mature leaf, flower and whole seedlings of the wild type *Oryza sativa* L. ssp. *Japonica*, *Nipponbare* plants using TRNzol reagent (TIANGEN, DP405-02) according to the manufacturer's instructions. RNAs were reverse transcribed using TransScript II reverse transcriptase (TransGen, AH301-02) to make the first strand of the complementary DNAs, as per manufacturer's instructions. These cDNAs were then used in the quantitative real time PCR (BIO-RAD, CFX96) using the gene specific primers; CX4699 (GACTCCTACTCCCACTTCGG) and CX4700 (GAGAACACTCAATCGCATAGACAT) for *OsPRMT1*, CX5008 (GGAACCCTCTTGGTGAATG) and CX5009 (GAAACGTAGGCGGAGAAAC) for *OsPRMT4*, CX4282 (GGGAAGATCAGTGAGTGGATT) and CX4283 (AGCATAGTTGGCACAAGAAGA) for *OsPRMT5*, CX4693 (AGTGCGCCAGAGATCCAAGAAG) and CX4694 (GATCTCTAGTAAAATATTTC) for *OsPRMT6a*, CX4695 (CTTCAAATCCCACAGAC) and CX4696 (CCGTGGTGCAATCTAGTTG) for *OsPRMT6b*, CX4697 (GCTATTATTTCATGGTGGGTACTTC) and CX4698 (GAGATGCTGGTTTGGTTATGTC) for *OsPRMT7*, and CX4284 (CGTCGAGGTGATACAGGG) and CX4285 (TAGCCACATCCGAGCAT) for *OsPRMT10*) to compare the expression of *OsPRMTs* in these tissues; the *ACTIN* gene (primers CX0909 (CCAATCGTGAGAAGATGACCCA) and CX0910 (CCATCAGGAAGCTCGTAGCTCT)) was used as internal control. Note: all the primer sequences given are in the 5^′^ to 3^′^ order.

### Construction of prokaryotic (*E.coli*) expression vectors

Constructs for prokaryotic expression of recombinant GST-fusion OsPRMT1, OsPRMT4, OsPRMT6a, OsPRMT6b, and OsPRMT10 were made by molecular cloning methods. Coding sequences of the OsPRMT1, OsPRMT4, OsPRMT6a, OsPRMT6b, and OsPRMT10 were amplified from the cDNAs of *Oryza sativa* L. ssp. *Japonica*, *Nipponbare* strain using the following primer pairs: CX0494 (CTCGAATTCATGGATCAGCGCAAGGGCAGCGGCAGCGAC) and CX0495 (CTCGAATTCTCTCATATCGGCAGGCCAGTTC) for GST-OsPRMT1, CX0500 (CGCGGATCCATGGCGTCGCCGGACCTGTTCCCGAAC) and CX 0501 (CTCGAATTCTAGTGAAGGCCGCCGAGTCTTG) for OsPRMT4, CX0496 (CGCGGATCCATGTTCGCCGGCGGCGCGGATGGCGGCAA) and CX0497 (CTCGAATTCACCTATCCCACCCTTGGTCGGC) for GST-OsPRMT6a, CX3254 (GTCGGATCCATGCTGCCGTCGCACCTCAAC) and CX3255 (GTGCTCGAGTCATCGCATGGCATAATCT) for GST-OsPRMT6b and CX0696 (ATAGGATCCATGGCGTCCCTCCCCAACGG) and CX0697 (CGCGAATTCGGTAGTATTACACAATGTGGCC) for GST-OsPRMT10. The PCR products were separated on 1% agarose gel, purified and digested with *EcoR*I (For OsPRMT1), *BamH*I and *EcoR*I (for OsPRMT4, OsPRMT6a and OsPRMT10), and *BamH*I and *Xho*I (for OsPRMT6b). Vector pGEX-4T-2 (Amersham Biosciences) was digested simultaneously with respective restriction enzymes and the digested and purified coding sequences of the OsPRMTs were ligated into it, to construct the *Escherichia coli* expression vectors. The GST-fusion OsPRMT5 *E. coli* expression vector was generated by the ligation independent cloning (LIC) strategy [43]; primers CX2492 ( TACTTCCAATCCAATGCGATGCCGCTGGGGCAACGCGCGGGGG) and CX2493 ( TTATCCACTTCCAATGCGCTATTATAGGCCGACCCAATATGATCG) were used to amplify the coding sequence of OsPRMT5.

In order to generate the MBP (maltose binding protein)-tagged OsPRMT6a, OsPRMT6a fragment amplified with the above stated primers and vector pMal-2×His-TE were digested with the *BamH*I and *EcoR*I and ligated with T4 DNA ligase (New England BioLabs, M0202S). Note: all the primers sequences given are in the 5^′^ to 3^′^ order.

### Construction of eukaryotic (plant) expression vectors

Binary vectors for the expression of GFP-fusion OsPRMT1, OsPRMT5, OsPRMT6a, OsPRMT6b, and OsPRMT10 in plant were made by following the conventional molecular cloning methods. Details of the primers are as: CX4334 (GGAAGATCTATGGATCAGCGCAAGGG) and CX4335 (GCGTCTAGAATCACGGGAACAAGCAC) for GFP-OsPRMT1, CX0500 and CX4336 (GCGTCTAGACTATCTAGTGAAGGCCGCC) for GFP-OsPRMT4, CX4337 (GTGCCATGGAGATGCCGCTGGGGCAACGCGC) and CX4338 (GTGCCATGGATAGGCCGACCCAATATGATC) for GFP-OsPRMT5, CX0496 and CX0497 for GFP-OsPRMT6a, CX3254 and CX4333 (GTGTCTAGATCATCGCATGGCATAATCT) for GFP-OsPRMT6b and CX0696 and CX4339 (GCGTCTAGACAAGCAAGGTTCAGACAT) for GFP-OsPRMT10. The purified PCR products and vector pCAMBIA1300 (CAMBIA, Canberra, Australia)-GFP were simultaneously digested with *Bgl*II and *Xba*I for GFP-OsPRMT1, *BamH*I and *Xba*I for GFP-OsPRMT4, GFP-OsPRMT6b and GFP-OsPRMT10, *Nco*I for GFP-OsPRMT5, and *BamH*I and *EcoR*I for GFP-OsPRMT6a. The digested *OsPRMTs'* fragments and vectors were ligated using T4 DNA ligase. Note: all the primers sequences given are in the 5^′^ to 3^′^ order.

### Tobacco (*Nicotiana benthamiana*) infiltration and fluorescent microscopy

The Agrobacterial strain (EHA105) transformed with binary vectors for the expression of GFP-fused OsPRMTs and P19 protein (suppressor of the RNAi silencing) were grown in the LB medium supplemented with selection antibiotics, acetosyringone (0.4 µM) and MES (10 mM). The cultures were centrifuged to pellet the Agrobacterial cells, resuspended in 10 mM magnesium chloride (MgCl_2_) and 0.2 mM acetosyringone and left to stand for 3 hours. The resuspended cultures harboring GFP-OsPRMTs and P19 construct were mixed in 1∶1 ratio and infiltrated into the 3-week-old healthy tobacco leaves by using syringes. The leaves were sectioned after 3 days of infiltration and the GFP signal was captured using fluorescence microscope (Olympus, Japan).

### Expression, purification and *in vitro* methyltransferase activity assay

The GST-fused OsPRMT1, OsPRMT4, OsPRMT5, OsPRMT6a, OsPRMT6b, and OsPRMT10 were expressed in *E. coli* expression strain, BL21, affinity purified by binding to the glutathione-Sepharose (GST) 4B beads (Amersham Biosciences, 17-0756-01). *In vitro* methyltransferase assays were performed as described previously [44]. The incubated proteins were separated by 15% SDS-PAGE, stained with Coomassie blue and visualized by fluorography.

### Western blot analysis

GST and GST-fused OsPRMT1, OsPRMT4, OsPRMT5, OsPRMT6b, and OsPRMT10 were incubated with the purified calf thymus core histones (Roche, 10223565001) in the presence of non-radioactive SAM at 30°C for 3 hours. The reaction mixtures were separated by the 15% SDS-PAGE, proteins were transferred to the PVDF membrane (Millipore, ISEQ00010) at 4°C and probed with antibodies; anti-H3R2 (Millipore, 05-808, rabbit monoclonal, 1∶3000) anti-H3R17me2a (Abcam, 8284, rabbit polyclonal, 1∶1500), anti-H4R3me2a (Millipore, 07-213, rabbit polyclonal, 1∶1000), anti-H3R4me2s (Abcam, ab5823, rabbit polyclonal, 1∶3000), and ASYM24 (Millipore, 07-414, rabbit polyclonal, 1∶1000) in TBST (0.1% Tween20, 150 mM NaCl and 50 mM Tris/HCl, pH 7.5)]. An equal amount of the reaction mixtures were separated by the 15% SDS-PAGE, followed by western blot analysis using anti-H3 (Millipore, 05-499, mouse monoclonal, 1∶2000) and anti-H4 (Millipore, 07-108, rabbit polyclonal, 1∶3000) antibodies in TBST buffer, for equal loading. Secondary antibodies used were mouse IgG HRP conjugate (Amersham Biosciences, NA931, 1∶40000) and rabbit IgG HRP conjugate (Amersham Biosciences, NA934, 1∶40000). The signal was detected on PVDF membrane with SuperSignal substrate (Thermo Scientific, 34076).

### *In vitro* interaction assay

Briefly; MBP-hexahistidine (HIS), GST, MBP-HIS-OsPRMT6a and GST-OsPRMT6b were expressed in *E. coli* and purified by affinity chromatography using GST beads (Amersham Biosciences, 17-0756-01) and HIS beads (Ni-charged resin, BIO-RAD, 156-0135). Bound proteins were eluted; about 2 µg was rebound to glutathione-Sepharose and HIS resins and used as affinity matrices. Roughly 2 µg of the resin bound proteins, GST and GST-OsPRMT6b, were used as bait to pull-down the same amount of eluted prey proteins, MBP-HIS and MBP-HIS-OsPRMT6a. The proteins mixtures were spun at 10,000 g for 1 minute, the resin was extensively washed with modified RIPA buffer (20 mM Tris/HCl, pH 7.5, 150 mM NaCl, 5 mM EDTA, 1% Nonidet P40 and 0.5% sodium deoxycholate, with 1 mM PMSF and 0.5 mM dithiothreitol (DTT), added immediately before use). The bound proteins were resuspended in 2× SDS loading buffer, boiled and separated by 10% SDS-PAGE. Proteins were transferred on to PVDF membrane (Millipore, ISEQ00010) and were probed with MBP antiserum (mouse monoclonal antiserum prepared in our lab, 1∶5000). The reciprocal pull-down experiment was performed similarly by using resin bound MBP-HIS and MBP-HIS-OsPRMT6a as bait to pull-down the eluted prey proteins GST and GST-OsPRMT6b and detected with the GST antiserum (mouse monoclonal antiserum prepared in our lab, 1∶30,000).

## Results

### *Oryza sativa* genome encodes eight homologs of the PRMTs

**Figure 1:**
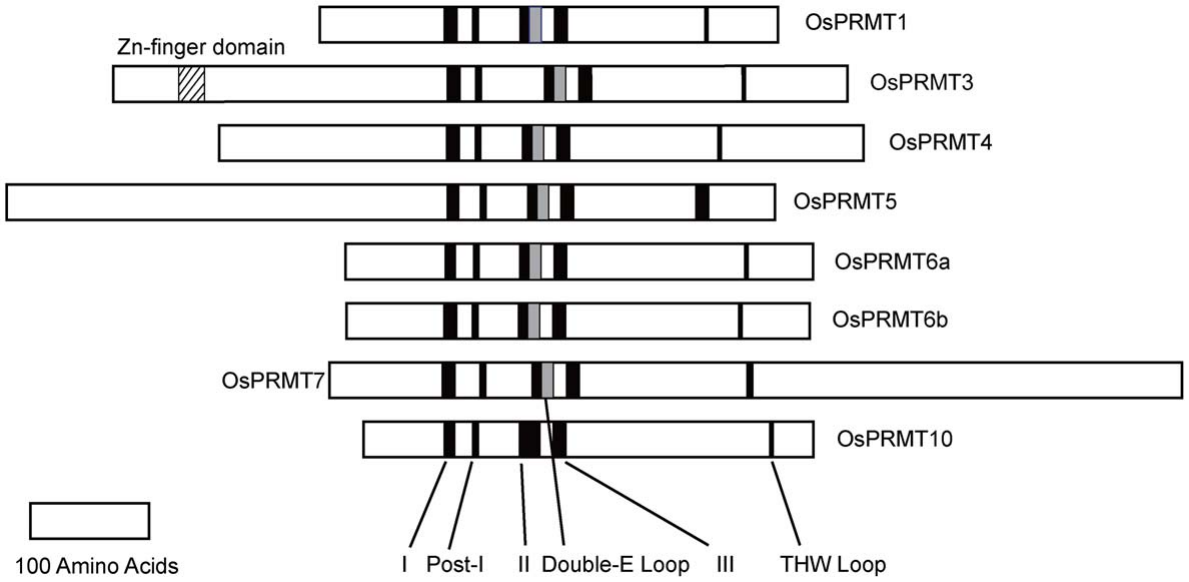
Members of the protein arginine methyltransferase family of proteins in *Oryza sativa*. Eight members of the *Oryza sativa* PRMT family; sharing the conserved PRMT signature motifs I, post-I, -II, -III, THW loop (Black bars) and double E loop (grey bars). PRMT3 harbors an additional Zn Finger domain (lined bar).

Eight loci, namely (according to TIGR/MSU database) Os09g19560 (NCBI accession NP_001062975.1), Os07g44640 (NCBI accession NP_001060419), Os07g47500 (NCBI accession NP_001060600), Os02g04660 (NCBI accession NP_00104584), Os10g34740 (NCBI accession NP_001064916), Os04g58060 (NCBI accession NP_001174145), Os06g01640 (NCBI accession NP_001056556), and Os06g05090 (NCBI accession NP_001056772) were found to encode putative *OsPRMTs*, previously, and were designated as *OsPRMT1*, *OsPRMT3*, *OsPRMT4*, *OsPRMT5*, *OsPRMT6a*, *OsPRMT6b*, *OsPRMT7* and *OsPRMT10*, respectively [42]. These eight genes coding for the OsPRMTs are distributed on six chromosomes i.e. 2, 4, 6, 7, 9, and10 (Figure S1). Here we aligned the amino acid sequences of these OsPRMTs to their Arabidopsis counterparts and found that OsPRMT1 shares 83% similarity to AtPRMT1a (and 86% to AtPRMT1b), OsPRMT3 shares 66% similarity to AtPRMT3, OsPRMT4 shares 85% similarity to AtPRMT4a (and 82% to AtPRMT4b), OsPRMT5 shares 82% similarity to AtPRMT5, OsPRMT6a shares 82% similarity to AtPRMT6, OsRMT6b share 79% similarity to AtPRMT6, OsPRMT7 shares 69% similarity to AtPRMT7 and OsPRMT10 shares 79% similarity to AtPRMT10 (Table S1). Multiple sequence alignment of the putative proteins expressed by these genes revealed a high degree of primary structure conservation in the PRMT signature motifs (I, post I, double E loop, post II, post III and THW (Thr-His-Trp)) loop (Figure 1), suggesting arginine methyltransferase activity for these proteins, as well as some additional sequences on both N- (OsPRMT3, OsPRMT4 and OsPRMT5) and C- (OsPRMT5 and OsPRMT7) terminals (Figures 1 and S2), which may be required for substrate specificity or some other still undetermined functions. The OsPRMT5 and OsPRMT7 sequences neither matched well with other OsPRMTs nor with each other (Figure S2) but when they were aligned to their Arabidopsis and human counterparts separately, they showed significant similarity in amino acid sequences (data not shown). In addition, *PRMT6* has two homologs sharing 58% identity and 72% similarity at amino acid level. The molecular weights of the putative expressed proteins are in the range of 42.72 to 89.72 kD (Table 1).

**Table 1:** Summary table.

	Tigr/IRGSP	RefSeq	Length	Molecular	Identity/	Subcellular	Tested	Methylation	Type of
	Locus	Accession	(aa)	Weight (D)	Similarity to	Localization	Substrates	Sites on	PRMT
					AtPRMT (%)			H3 and H4	
**OsPRMT1**	Os09g19560/	NP_001062975	387	43013.9	73/83, 77/86	N and C	GAR, Myelin	H3R17and	I
	Os09g0359800						Basic Protein,	H4R3	
							H4, H2A, H3		
**OsPRMT3**	Os07g44640/	NP_001060419	620	67942.5	51/66	ND	ND	ND	ND
	Os07g0640000								
**OsPRMT4**	Os07g47500/	NP_001060600	545	60108.9	73/85, 70/82	ND	Myelin Basic	H3R17	I
	Os07g0671700						Protein and H3		
**OsPRMT5**	Os02g04660/	NP_001045843	649	72673.1	71/82	N and C	Myelin Basic	H4R3	II
	Os02g0139200						Protein, GAR,		
							H4, H2A		
**OsPRMT6a**	Os10g34740/	NP_001064916	395	43847.4	64/82	Mainly N	GAR	ND	ND
	Os10g0489100								
**OsPRMT6b**	Os04g58060/	Q7XKCO.2*	392	43518.6	62/79	Mainly N	Myelin Basic	H3R2 and	I
	Os04g0677066						Protein, GAR,	R17	
							H3, H4, H2A		
**OsPRMT7**	Os06g01640/	NP_001056556	721	80600.8	54/69	ND	ND	ND	ND
	Os06g0105500								
**OsPRMT10**	Os06g05090/	NP_001056772	381	42717.5	64/79	N and C	Myelin Basic	H3R2 and	I
	Os06g0142800						Protein, GAR,	H4R3	
							H4, H2A, H3		

Abbreviations: ND, Not Determined; N, Nucleus; C, Cytoplasm; H3, histone H3; H4; histone H4; H3R2, histone H3 arginine 2; H3R17, histone H3 arginine 17; H4R3, histone H4 arginine 3; rest of the abbreviations are expanded elsewhere in the paper. TIGR/MSU loci were taken from http://rice.plantbiology.msu.edu/ and IRGSP (Japonica group) loci were taken from NCBI website http://www.ncbi.nlm.nih.gov/.

*is the Swiss-prot identifier for OsPRMT6b protein. The no of amino acids and molecular weights of the proteins are taken from NCBI http://www.ncbi.nlm.nih.gov.

### *OsPRMT* genes reveal distinct expression patterns

**Figure 2:**
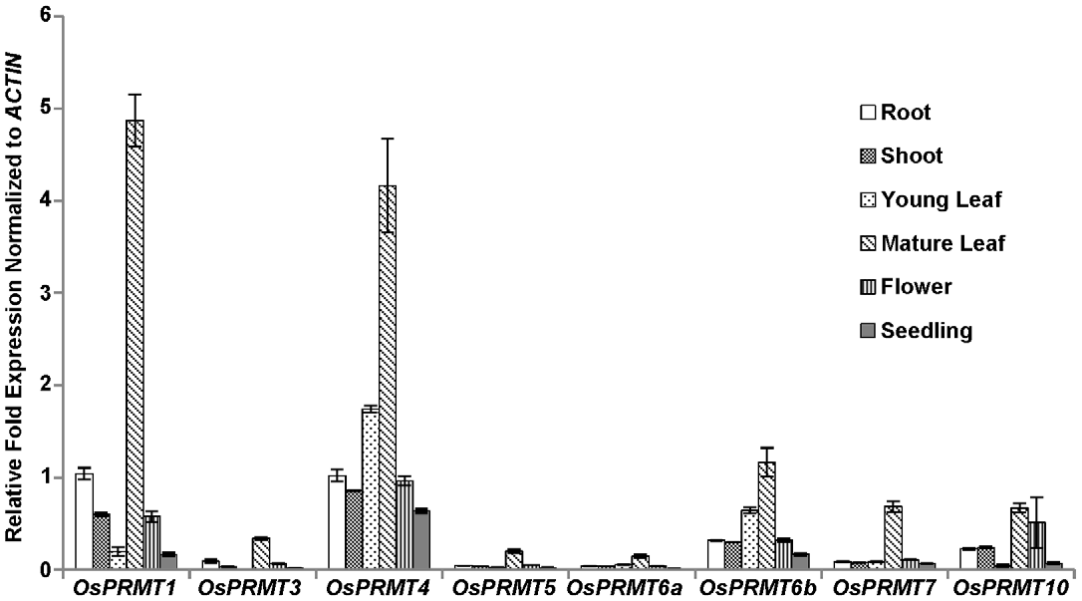
Relative expression patterns of the *OsPRMTs*. Total RNAs were isolated from plant tissues; root, young leaf, mature leaf, shoot, flower and whole seedling of the *Nipponbare* strain and cDNAs were synthesized. The relative fold change in the expression of the *OsPRMT1*, *OsPRMT3*, *OsPRMT4*, *OsPRMT5*, *OsPRMT6a*, *OsPRMT6b*, *OsPRMT7* and *OsPRMT10* was normalized to the expression of *ACTIN*. The error bars represent standard deviation.

In order to illustrate the spatial expression patterns of the *OsPRMTs*, total RNAs were isolated from root, flower, shoot, young leaf, mature leaf and whole seedlings. These RNAs were reverse transcribed to cDNAs and analyzed by quantitative real time PCR to check the expression of the *OsPRMTs* in these tissues, using specific primer pairs designed for this purpose, as described in the experimental part. As shown in figure 2, *OsPRMTs* display distinct expression patterns in a tissue specific manner. Among all the *OsPRMTs*, *OsPRMT1* and *OsPRMT4* are expressed relatively higher than other homologs in all the tissue types analyzed in this study, which is consistent with their involvement in a variety of biological processes as studied in animals and yeast. Next in the relative abundance are *OsPRMT6b* and *OsPRMT10*. However, among the tissue types analyzed in this study, the mature leaf contains high abundance of all the *PRMTs*. *OsPRMT6b* and *OsPRMT10* also exhibit significant expression in the root, shoot, young leaf and flower, the significance of which awaits further investigation. *AtPRMT10* has been implicated in regulation of important traits in Arabidopsis such as flowering time and the *PRMT6* in human has been reported to involve in transcriptional regulation and antiviral defense; however its role in plants remains to be unearthed. *OsPRMT6b* is expressed more than its homolog, *OsPRMT6a*, which is consistent with our *in vitro* enzymatic assay which shows that only the OsPRMT6b shows strong ability to catalyze the transfer of methyl group, whereas OsPRMT6a has very weak activity.

### OsPRMT1, OsPRMT4, OsPRMT5, OsPRMT6b and OsPRMT10 have *bona fide* methyltransferase activity *in vitro*

**Figure 3:**
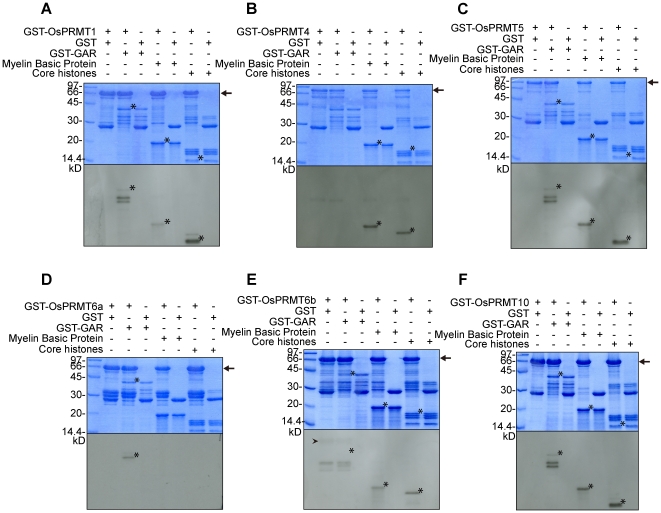
*In vitro* methyltransferase activity assay of the OsPRMTs. Methyltransferase activity assay of OsPRMT1 (A), OsPRMT4 (B), OsPRMT5 (C), OsPRMT6a (D), OsPRMT6b (E), and OsPRMT10 (F). GST and GST-OsPRMTs were bound to the GST beads and incubated with indicated substrates; myelin basic protein, GST-GAR and calf thymus core histones in the presence of [^3^H]SAM for 3 hours in a final volume of 30 µl of HMT buffer (20 mM Tris/HCl, 4 mM sodium EDTA, 1 mM PMSF and 1 mM dithiothreitol). Methylated proteins were separated by 10% SDS-PAGE, stained with Coomassie blue (upper panels), destained, soaked in amplify (Amersham Biosciences, NAMP100) dried and visualized by fluorography by exposing to the x-ray film at −80°C for 48 hours (lower panels). The corresponding proteins are indicated above the upper panels. The arrow “→” points GST-Tagged full length OsPRMTs whereas the asteric “_*_” represents the substrate proetins i.e. GST-GAR, myelin basic protein and core histones (H3, H4, H2A and H2B). The arrow head “
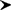
” indicates the automethylation of OsPRMT6b.

The conservation of the PRMT signature motifs in the OsPRMTs prompted us to decipher their *in vitro* arginine methyltransferase activity. For this purpose, GST and GST-fusion full length OsPRMT1, OsPRMT4, OsPRMT5, OsPRMT6a, OsPRMT6b, and OsPRMT10 were expressed in *E. coli* and affinity purified with the glutathione-Sepharose 4B (GST) beads. These proteins were assayed for *in vitro* methyltransferase activity in the presence of radioactive SAM ([^3^H]SAM) and the substrate proteins; GST-GAR, myelin basic protein and purified calf thymus core histones. The reaction mixtures were separated by 15% SDS-PAGE and visualized by fluorography (Figures 3 and S3). The exposure time for figure 3 and figure S3 is 48 hours and4 days, respectively. The GST-fusion OsPRMTs transferred ^3^H-labeled CH_3_-group onto the substrate proteins whereas the GST alone could not, which was used as negative control (Figures 3A–F and S3A–F). OsPRMT1 methylated GST-GAR and myelin basic protein; however, among the core histones H4 is methylated preferentially whereas histone H2A and H3 are weakly methylated, consistent with the earlier reports in human and Arabidopsis (Figures 3A and S3A). Like PRMT4/CARM1 in animals, OsPRMT4 did not methylate GST-GAR, however it methylated myelin basic protein and histone H3 (Figures 3B and S3B). Similar to the Arabidopsis counterpart [36], OsPRMT5 methylated histone H4, GST-GAR, and myelin basic protein; and to some extant H2A (Figures 3C and S3C). OsPRMT6a displayed activity for GST-GAR but none of the other substrates were methylated; showing that it either does not methylate the myelin basic protein and core histones or it has weak activity (Figures 3D and S3D). OsPRMT6b showed relatively stronger activity and methylated GST-GAR, myelin basic protein and histone H3 (Figure 3E). However, it also shows activity against H4 which is visible after longer exposure (Figure S3E). Additionally, OsPRMT6b showed automethylation like its human counterpart, as indicated by the arrow head (Figures 3E and S3E). As depicted by figure 3F, OsPRMT10 catalyzed the methylation of GST-GAR, myelin basic protein, and H4 (in accordance with the Arabidopsis PRMT10 [42]) (Figure 3F), and to a little extant H3 and H2A which is evident after four days exposure (Figure S3F). There are some non-specific bands, especially in the longer exposure films which probably show methylation of the degradation products of the enzymes and/or substrates (Figure S3). These results indicate that OsPRMT1, OsPRMT4, OsPRMT5, OsPRMT6b, and OsPRMT10 are *bona fide* arginine methyltransferases *in vitro*.

### OsPRMT1, OsPRMT4, OsPRMT5, OsPRMT6b and OsPRMT10 methylate specific arginine residues on histone H3 and H4 and can be classified into two types of PRMTs

**Figure 4:**
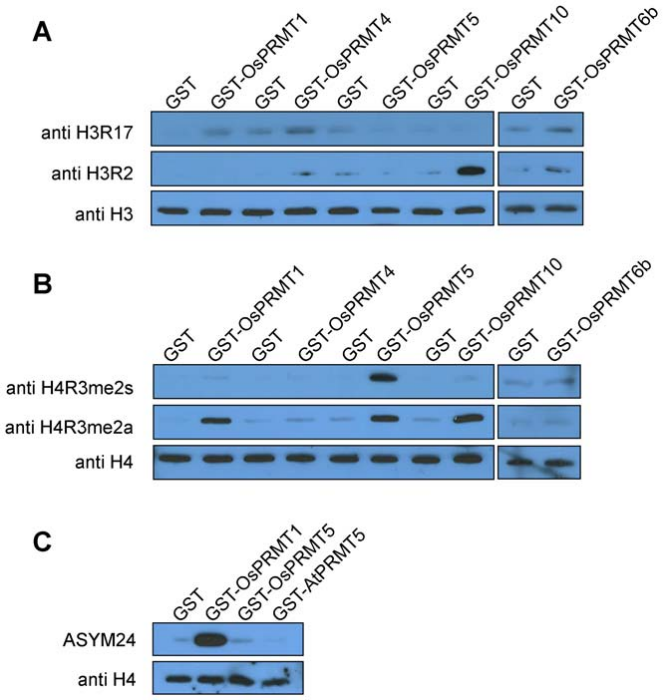
Site specificities of the OsPRMT1, OsPRMT5, OsPRMT6b, and OsPRMT10. (A, B and C) Purified calf thymus core histones were either incubated with negative control, GST or GST-OsPRMT1, GST-OsPRMT5, GST-OsPRMT6b, GST-OsPRMT10 and GST-AtPRMT5 and were separated by 15% SDS-PAGE, followed by western blot analysis using anti-H3R2me2a, anti-H3R17me2a, anti-H4R3me2a, anti-H4R3me2s, and ASYM24 antibodies. An equal amount of the reaction mixtures were separated by the 15% SDS-PAGE, followed by western blot analysis using anti-H3 and anti-H4 antibodies, for equal loading (lowest panels in A, B and C). The antibodies are indicated to the left of each panel, whereas the corresponding PRMTs are represented above the upper panels.

Different PRMTs have distinct specificities for the methylation sites in the histone tails which in turn contribute to the histone code (as reviewed in [45]). In order to investigate which particular arginine residues are methylated by each OsPRMT analyzed in calf thymus core histones, western blot analysis was performed after the *in vitro* methylation reaction using antibodies against different methylated arginine residues. Calf thymus core histones incubated with GST protein served as negative control. The methylated substrates detected by antibodies against aDMA were considered to be methylated by type I PRMT whereas those detected by antibodies against sDMA were thought to be methylated by type II PRMT. OsPRMT1 asymmetrically dimethylated H3 at R17 and H4 at R3 hence it belongs to type I PRMTs (Figures 4A and 4B, left panels). OsPRMT4 methylated H3R17 which is consistent with the earlier reports and it belongs to type I PRMT because its substrate was detected by antibody against asymmetrically dimethylated H3R17 (Figures 4A and 4B, left panels). OsPRMT5 belongs to type II PRMTs, since the substrates methylated by it were recognized by the antibody against sDMA and it symmetrically dimethylated R3 in histones H4 (Figures 4A and 4B, left panels), which is in consistence with our previous study on Arabidopsis PRMT5 [36]. However, unexpectedly the histone H4 incubated with OsPRMT5 was also detected by the antibody against aDMA at H4R3 which is probably due to the ability of the antibody to detect both the symmetric as well as the asymmetric dimethyl H4R3. To address this problem, the histone H4 incubated with the GST, GST-OsPRMT1, GST-OsPRMT5 and GST-AtPRMT5 (as control) were probed with anti-aDMA (ASYM24) antibody. The ASYM24 antibody detected the arginine methylated product from OsPRMT1 but not from the OsPRMT5 and AtPRMT5 (Figure 4C), which proves that OsPRMT5 is indeed a type II PRMT catalyzing the formation of MMA and sDMA in the substrate proteins. OsPRMT10 is a type I PRMT because it asymmetrically dimethylates H3 at R2 and H4 at R3 (Figures 4A and 4B, left panels). OsPRMT6b is a type I PRMT and it asymmetrically dimethylates H3 at R2 and 17 (Figures 4A and 4B, right panels). From the *in vitro* enzymatic activity assay it is evident that OsPRMT6b has relatively stronger activity at histones H3 and H4 (Figure 3E and Figure S3E), but here we did not see any activity at H4R3 which shows that it might be methylating some other arginine residue(s) in histone H4, which awaits further investigation.

### OsPRMT6a interacts with OsPRMT6b and inhibits its methyltransferase activity *in vitro*

**Figure 5:**
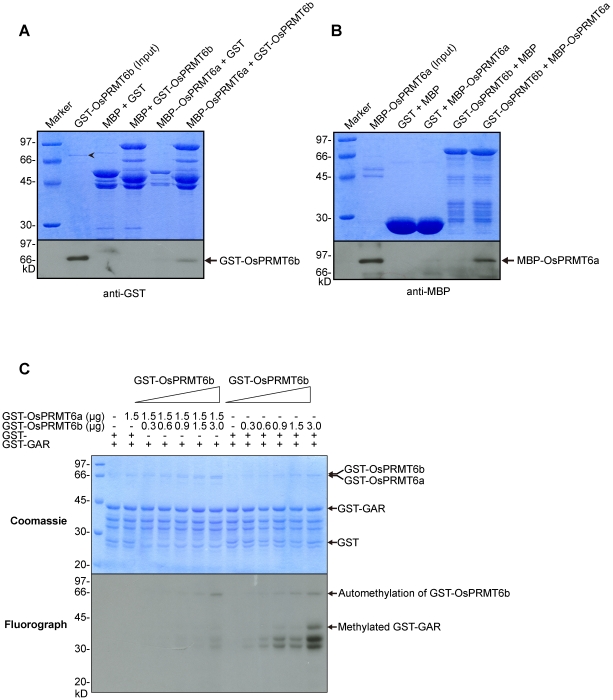
OsPRMT6a and OsPRMT6b directly interact *in vitro*. (A and B) GST, MBP, MBP-OsPRMT6a and GST-OsPRMT6b were expressed in *E. coli*. Reciprocal pull-down assay was performed as described in the experimental part. Equal amount of each mixture of proteins was separated on two separate 10% SDS-PAGE, one for Coomassie staining (upper panels A and B) for equal loading and the other for western blotting (lower panels A and B). GST and MBP alone were used as negative controls. The protein pairs are indicated at the top of the upper panels. MBP-OsPRMT6a served as bait and GST-OsPRMT6b as prey (A) and *vice versa* (B). A 10 µl cell extract from *E. coli* containing MBP-OsPRMT6a and GST-OsPRMT6b was used as input. Western blotting using GST and MBP monoclonal antisera revealed the interaction between OsPRMT6a and OsPRMT6b. The arrow head “???” indicates MBP-OsPRMT6a. (C) 0.3, 0.6, 0.9, 1.5 and 3.0 µg of GST-OsPRMT6b was mixed either with 1.5 µg of GST-OsPRMT6a or with GST protein alone (as control) in an *in vitro* methyltransferase activity assay as described in figure 3. The Coomassie blue stained and dried gel was exposed at −80°C for four days.

Rice has two homologs of the PRMT6, OsPRMT6a and OsPRMT6b. Our *in vitro* enzyme activity assay showed that OsPRMT6b has strong activity but the OsPRMT6a has weak activity only against the GAR domain (Figures S3D and S3E), which indicates that OsPRMT6a has either lost its methyltransferase activity or acquired another function during the course of evolution. Since the PRMTs harbor a characteristic dimerization arm, which is considered as essential for the enzymatic activity [46], we checked if OsPRMT6a and OsPRMT6b could form a heterodimer *in vitro*. For this purpose we expressed the GST, MBP-HIS, GST-OsPRMT6a, MBP-HIS-OsPRMT6a, GST-OsPRMT6b, and MBP-HIS-OsPRMT6b in *E. coli* and performed the reciprocal pull-down experiments to check their direct interaction *in vitro*. In the first experiment we used the resin bound ‘bait’ proteins, MBP-HIS (as negative control) and MBP-HIS-OsPRMT6a to pull down the ‘prey’ proteins, GST (as negative control) and GST-OsPRMT6b (Figure 5A). In the reciprocal experiment the resin bound ‘bait’ proteins GST (as negative control) and GST-OsPRMT6b were used as to pull-down the ‘prey’ proteins, MBP (as negative control) and MBP-HIS-OsPRMT6a (Figure 5B). The proteins were separated on two 10% SDS-PAGE, one was stained with Coomassie blue for equal loading and other was used for western blot. The monoclonal anti-GST (Figure 5A) and anti-MBP antibodies (Figure 5B) were used to detect the GST, GST-OsPRMT6b, MBP-HIS and MBP-HIS-OsPRMT6a proteins. As expected, in the reciprocal pull-down experiment we found that OsPRMT6a and OsPRMT6b directly interact with each other *in vitro* but not with the GST and MBP-HIS and they may form a heterodimer *in vivo*. Further to explore the significance of this interaction, GST-OsPRMT6a was mixed with different amounts of GST-OsPRMT6b in an *in vitro* methyltransferase assay where GST-GAR was used as substrate. In control reaction the same amount of GST-OsPRMT6b was mixed with only GST protein. Interestingly, GST-OsPRMT6a but not the GST protein inhibited the *in vitro* methyltransferase activity of the GST-OsPRMT6b (Figure 5C). Since, dimerization is essential for the enzymatic activity of the PRMTs, this finding reveals a novel mechanism of regulation of PRMTs' activity, probably, by heterodimerization.

### Subcellular localization of the OsPRMT1, OsPRMT4, OsPRMT5, OsPRMT6a, OsPRMT6b, and OsPRMT10

**Figure 6:**
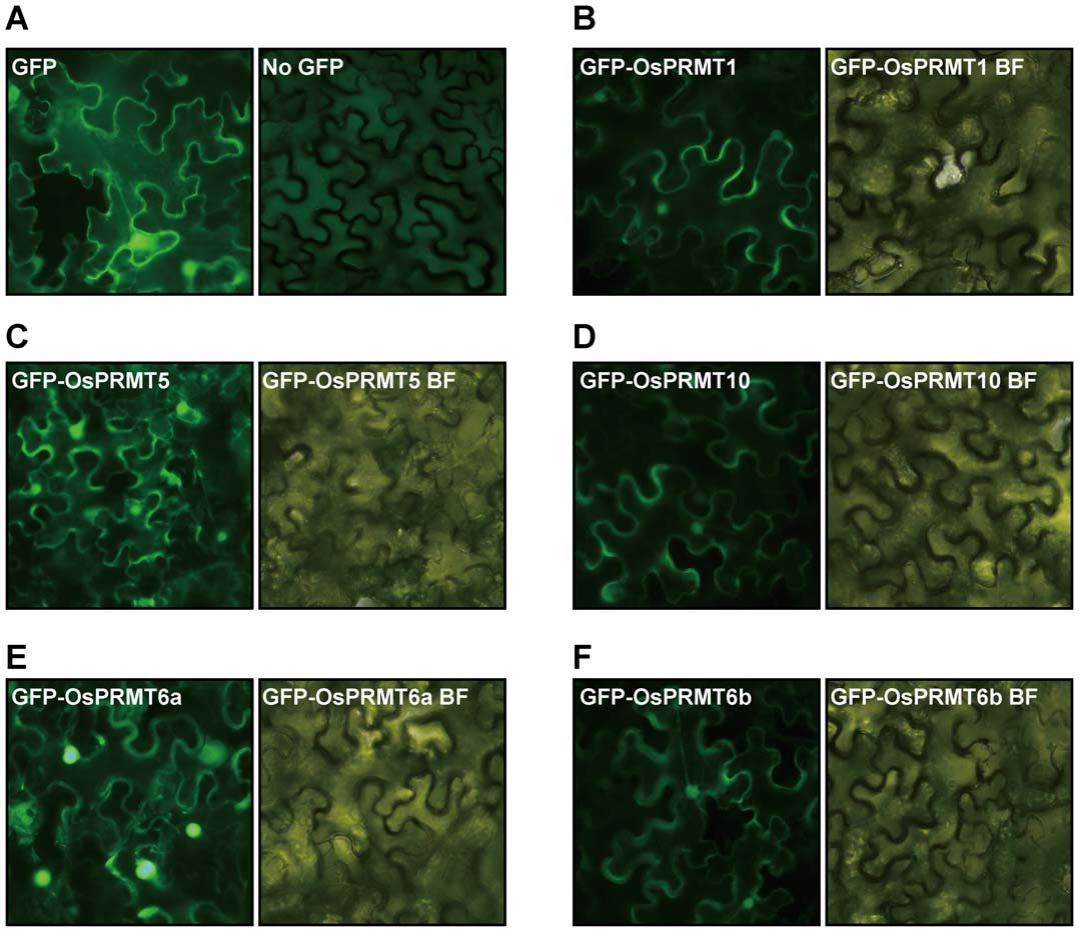
Subcellular localization of the OsPRMT1, OsPRMT5, OsPRMT6a, OsPRMT6b, and OsPRMT10. OsPRMT1, OsPRMT5, OsPRMT6a, OsPRMT6b, and OsPRMT10 were fused in frame to GFP in binary vector, transformed to the Agrobactrial strain, EHA105 and injected into the *Nicotiana benthamiana* leaves. Expression of GFP and GFP-tagged OsPRMT1, OsPRMT5, OsPRMT6a, OsPRMT6b, and OsPRMT10 was observed under fluorescence microscopy and bright field microscopy (designated by BF) after 2–3 days.

Since OsPRMTs methylate a variety of substrates such as histones (nuclear basic proteins), GAR domain of Arabidopsis fibrillarin (an RNA binding nucleolar protein) and myelin basic protein (a cell membrane protein); they may localize to and function in different compartments of the cell. To find out the specific subcellular localization of the OsPRMTs, the coding sequences of the OsPRMT1, OsPRMT5, OsPRMT6a, OsPRMT6b, and OsPRMT10 were fused in frame to the GFP, driven by the *CMV* 35S promoter and transfected into the *Nicotiana benthamiana* leaves by the Agrobacterial strain EHA105. After 3 days of injection, the GFP signal was checked under the fluorescence microscope. GFP alone (used as control) was localized both to the nucleus as well as to the cytoplasm (Figure 6A). OsPRMT1, OsPRMT5, and OsPRMT10 exhibited the same subcellular location both in the cell nucleus and cytoplasm (Figures 6B–6D), which is consistent with the localization of their Arabidopsis and human counterparts. However, the OsPRMT6a and OsPRMT6b were mainly localized into the nucleus (Figures 6E and 6F), which is also in agreement with the localization of human PRMT6 [47]. The localization of the OsPRMTs into both the cytoplasm and nucleus designates their general requirement in many basic cellular processes.

## Discussion

The evolutionary conservation of arginine methylation from unicellular eukaryotes to plants and humans indicates its involvement in the basic cellular machinery. *Saccharomyces cerevisiae* genome encodes three PRMTs (HMT1, Hsl7 and Rmt2) [12], Drosophila genome contains 9 (DART1 to DART9) [48], Arabidopsis genome bears 9 (*AtPRMT1a*, *AtPRMT4b*, AtPRMT3, *AtPRMT4a*, *AtPRMT4b*, *AtPRMT5*, *AtPRMT6*, *AtPRMT7*, and *AtPRMT10*) [42] and human genome bears 11 (*hPRMT1*-*11*) [1]; as known, so far. PRMTs have been studied extensively in animals and yeast. However, in the recent years there is a growing tendency to substantiate their functional importance in plants, especially in the model plant *Arabidopsis thaliana* [33], [35]–[38], [40]–[42]. *PRMT1*, *PRMT3* and *PRMT5* have been found to be strictly conserved during the evolution of eukaryotes from unicellular yeasts, molds, amoebae, and protozoa to higher plants and animals; however, the other PRMTs are less conserved which might have evolved in the multicellular organisms as requirement for tissue specific functions. Consistent with this hypothesis, *PRMT2* and *PRMT8* exhibit tissue specific expression [49], [50]. Additionally, no homologs of *PRMT2* and *PRMT8* exist in the Arabidopsis and rice genomes which further strengthen the notion that these *PRMTs* arose for the tissue specific functions in higher eukaryotes.

Gene duplication is common phenomenon in eukaryotes which occurs at a rate of 0.01 per gene per million years and is a source of evolutionary novelties [51]. The duplication of gene may result in nonfunctionalization (one copy of gene is silenced), neofunctionalization (one copy acquire a new function) or subfunctionalization (both copies become partially compromised). Unlike animals and yeast, there are two homologs each of PRMT1 and PRMT4 in Arabidopsis and PRMT6 in rice. It seems that in Arabidopsis, duplication of PRMT genes, *AtPRMT1* and *AtPRMT4*, resulted in subfunctionalization, because the duplicated genes showed same expression patterns and methyltransferase activities. In addition, the double knockout mutant of *AtPRMT4a* and *AtPRMT4b* results in more severe phenotype than that of single knockout of these two copies of the gene [35]. In Oryza; although the expression pattern and cellular location of OsPRMT6a and OsPRMT6b are similar, OsPRMT6a almost lost methyltransferase activity, which suggests a nonfunctionalization outcome of OsPRMT6 duplication. Our data also suggest that OsPRMT6b may be regulated by OsPRMT6a which points to neofunctionalization; however, more solid evidence, such as the resolution of crystal structure of the heterodimer of the OsPRMT6a and OsPRMT6b is needed to support this idea.

The significance/functional importance of the *PRMTs* is evident from the quantitative real time PCR analysis data of the mRNAs from different tissues, revealing that they are widely expressed in many tissues, rather than limited to specific tissues/cell types. Among the tissue types studied; root, shoot, young leaf, mature leaf, flower and total seedling, mature leaf has highest pool of all the OsPRMTs (the physiological meaning of which awaits further investigation), whereas among the *OsPRMTs*, *OsPRMT1* and *OsPRMT4* are more abundant in all the tissue types. Additionally, the *in vivo* targeting of the OsPRMT1, OsPRMT4, OsPRMT5, and OsPRMT10 to both the nucleus and cytoplasm indicates the redundancy and more general requirement for these PRMTs; however, the predominant nuclear localization of the OsPRMT6a and OsPRMT6b reflects somewhat specificity for these enzymes. Further studying their expression pattern by the promoter-genomic-GUS analysis will hint their probable function in specific tissues and cells in more detail.

Our *in vitro* enzymatic activity assay demonstrated that OsPRMT1, OsPRMT4, OsPRMT5, OsPRMT6b, and OsPRMT10 can directly catalyze the transfer of methyl group from SAM to their substrate proteins and no additional cofactor(s), partner molecule or conformation is required for this activity. OsPRMT6a showed weak methyltransferase activity which suggests that it might have some additional requirement for its activity. Another possibility is that, this protein harbors the core PRMT motifs probably for some other unknown functions. This notion is strengthened by our *in vitro* titration experiment in which the addition of GST-OsPRMT6a inhibits the methyltransferase activity of GST-OsPRMT6b. However, further studies dedicated to finding the effect of making different combinations of the PRMTs on the methyltransferase activity of these PRMTs and resolving the crystal structures of heterodimers will give the conclusive evidence to this phenomenon. In the *in vitro* enzyme activity assay we used myelin basic protein, GST-GAR and core histones from the calf thymus as substrates for the OsPRMTs. Myelin basic protein is widely used as substrate for the enzymes responsible for post-translational modifications because it readily undergoes various posttranslational modifications such as phosphorylation, ubiquitination and methylation [52]. *In vivo*, the modifier enzymes affect its structure and, consequently, interactions with other membrane components in the membrane assembly which is a predominantly post-synthetic phenomenon. Arginine methylation of the myelin basic protein results in its compact structure; antagonizes the effect of citrolination by peptidylarginine deiminases (PADs), *in vivo* dimerization, and resistance to trypsin digestion. OsPRMT1, OsPRMT4, OsPRMT5, OsPRMT6b and OsPRMT10 displayed significant activity against the myelin basic protein in our methyltransferase assay, suggesting that these enzymes may methylate myelin basic protein *in vivo* and modulate the above stated as well as some yet unknown properties of this biologically important protein. GAR domains, characterized by the tri-peptide motif, RGG, are frequently found in RNA binding proteins including, nucleolar protein 1 (Nop1), Nop3, Npl3, human DNA 35 helicase II, ewing sarcoma protein, hnRNP A1, hnRNP A2, human MRE11, nucleolin, and fibrillarin. The structural integrity of GAR is required for the nucleic acid and protein binding, intracellular targeting and hence proper functioning of the proteins harboring this domain. The GAR domain of hnRNP A1 is required for cooperative binding to RNA, but it is also required for interactions with other pre-mRNA binding proteins. Nucleolin's GAR domain shows a specific interaction with ribosomal protein L3, thus strengthening a possible role in protein-protein interactions and perhaps assembly of ribosomal proteins into ribosome. AtPRMT1a and AtPRMT1b have been reported to directly interact with and methylate the fibrillarin protein in the GAR domain [33]. Here we outlined that all the tested OsPRMTs; OsPRMT1, OsPRMT4, OsPRMT5, OsPRMT6a, OsPRMT6b, and OsPRMT10 methylate the GAR domain *in vitro*. PRMT6 has two homologs in rice, namely OsPRMT6a and OsPRMT6b. OsPRMT6b has strong *in vitro* methyltransferase activity against myelin basic protein, GAR and histones H2A, H3 and H4; however, OsPRMT6a shows only weak activity against GAR domain, which shows that OsPRMT6b is the functional enzyme, whereas OsPRMT6a might has lost its activity during the course of evolution or it requires some additional cofactor or partner for its methyltransferase activity. Another possible explanation for the lack of enzymatic activity of OsPRMT6a is due to the requirement of hetero-dimerization with OsPRMT6b. Our *in vitro* pull-down experiment proved that they directly interact with each other. Taken together, our data implies that OsPRMTs may redundantly methylate the GAR domain in a wide variety of proteins, involved in a multitude of essential biological processes. Nonetheless, studying the plants with lesions in these genes and especially multiple mutants might be very handy to unveil the biological significance of arginine methylation in regulating plant development.

Histones are basic proteins and essential component of the chromatin, which carries the genetic information from one generation to other generation. Histone tails are prone to many post translational modifications (phosphorylated, acetylated, methylated, ubiquitinated, *etc*) at various amino acid residues such as lysine, serine, arginine, dicarboxylic acid *etc*; which in turn constitute the histone code. Different combinations of these modifications have distinct transcriptional outcomes. Among these modifications is the methylation of arginine in histone tails, both in animals and plants; Thus far, the major known sites liable to arginine methylation are R2, R8, R17, R26 of histone H3, and R3 of histone H4 [45]. We have recently summarized that how the epigenetic changes, especially chromatin modifications affect the plant development [45], [53]. In mammals, PRMT1 and PRMT5 asymmetrically and symmetrically dimethylate H4R3, respectively and are required for the normal development [6]. Arabidopsis PRMT1a and PRMT1b were reported to asymmetrically dimethylate H4R3 *in vitro* but its functional importance is yet to be discovered [33]. Furthermore, AtPRMT5/Skb1-mediated symmetric dimethylation at H4R3 regulates flowering time by repressing *FLC* transcription [36]. Our *in vitro* methyltransferase activity assay and western blot data consistently suggest that the OsPRMT1 and OsPRMT10 asymmetrically and OsPRMT5 symmetrically dimethylate H4R3. PRMT6-mediated asymmetric dimethylation at H3R2 antagonizes the H3K4 tri-methylation in mammals, which is a well-known transcriptional activation mark [54]. AtPRMT4a and AtPRMT4b, the Arabidopsis homologs of human PRMT4/CARM1, asymmetrically dimethylate R2, R17, and R26 in histone H3 *in vitro* and are required for the *in vivo* H3R17me2a and *atprmt4a atprmt4b* double mutant displays *FLC*-dependent late flowering phenotype [35]. In addition, asymmetric dimethylation at H4R3 catalyzed by AtPRMT10 regulates the Arabidopsis flowering time in an *FLC*-dependent manner. *In vitro* enzymatic activity assay combined with the immunoblotting experiment, in this study, proved that OsPRMT1, OsPRMT4 and OsPRMT6b asymmetrically dimethylated H3 at R17 whereas OsPRMT10 and OsPRMT6b methylated H3 at R2. In addition, OsPRM1 and OsPRMT10 also asymmetrically dimethylate H4R3, OsPRMT5 catalyzes symmetric dimethylation at this site. This *in vitro* data manifests redundancy for the methylation at these sites in H3 and H4; nevertheless, investigating the *in vivo* global changes in the methylation levels at these sites of histones, isolated from knockout mutants (single as well as multiple) plants, would probably uncover the specificities of OsPRMTs, which may require some proper conformation of the histones, for activity at these sites. Furthermore, unveiling the physiological importance of these conserved methylation marks also awaits further investigation of the mutant plants with lesion in the genes coding for these enzymes. In the nut-shell; this study strongly suggests the conservation of this important posttranslational modification of proteins, arginine methylation, in monocots and will surely trigger the understanding of biological significance of protein arginine methylation in the important cereal, *Oryza sativa*, and provide additional challenges and surprises about this evolutionarily conserved posttranslational modification event.

## Supporting Information

Figure S1 **Chromosomal distribution of OsPRMTs.** Chromosomal distribution of *PRMT* gene family member in *Oryza sativa*. Eight PRMT genes are distributed throughout the Oryza genome as single genes. Gene positions on the physical map of NCBI mapviewer are indicated in megabases for each gene. Six chromosomes on which eight *PRMT* genes localize are indicated by numbers (2, 4, 6, 7, 9, and10).
(TIF)

Figure S2 **Amino acid sequence alignment of the PRMT gene family members in** ***Oryza sativa*** **.** The TIGR/MSU identifiers for OsPRMT1, OsPRMT3, OsPRMT4, OsPRMT5, OsPRMT6a, OsPRMT6b, OsPRMT7 and OsPRMT10 are Os09g19560, Os07g44640, Os07g47500, Os02g04660, Os10g34740, Os04g58060, Os06g01640, and Os06g05090, respectively. Identical amino acids are boxed in black and similar amino acids are boxed in grey. Characteristic methyltransferase motifs, I, post-I, post-II, post-III are underlined, whereas Double E loop and THW loop are enclosed in boxes.
(TIF)

Figure S3 ***In vitro*** **methyltransferase activity assay of the OsPRMTs (longer exposure time).** S3A–S3F represent the *in vitro* enzyme activity assay result for the OsPRMT1, OsPRMT4, OsPRMT5, OsPRMT6a, OsPRMT6b and OsPRMT10, respectively. All the steps are essentially the same as in figure 3, however, the exposure time is four days.
(TIF)

Table S1 **Table representing the identities/similarities of OsPRMTs to the Arabidopsis PRMTs at amino acids level.** Amino acid sequences of the OsPRMTs and AtPRMTs were aligned in the bl2seq tool on the NCBI website to find the percent identity/similarity.
(DOC)
