# Supplementary figures and images for: Characterization of the PRMT Gene Family in Rice Reveals Conservation of Arginine Methylation
Source: PLoS One. 2011 Aug 11;6(8):e22664. doi: 10.1371/journal.pone.0022664 (PMC3154905; doi:10.1371/journal.pone.0022664)

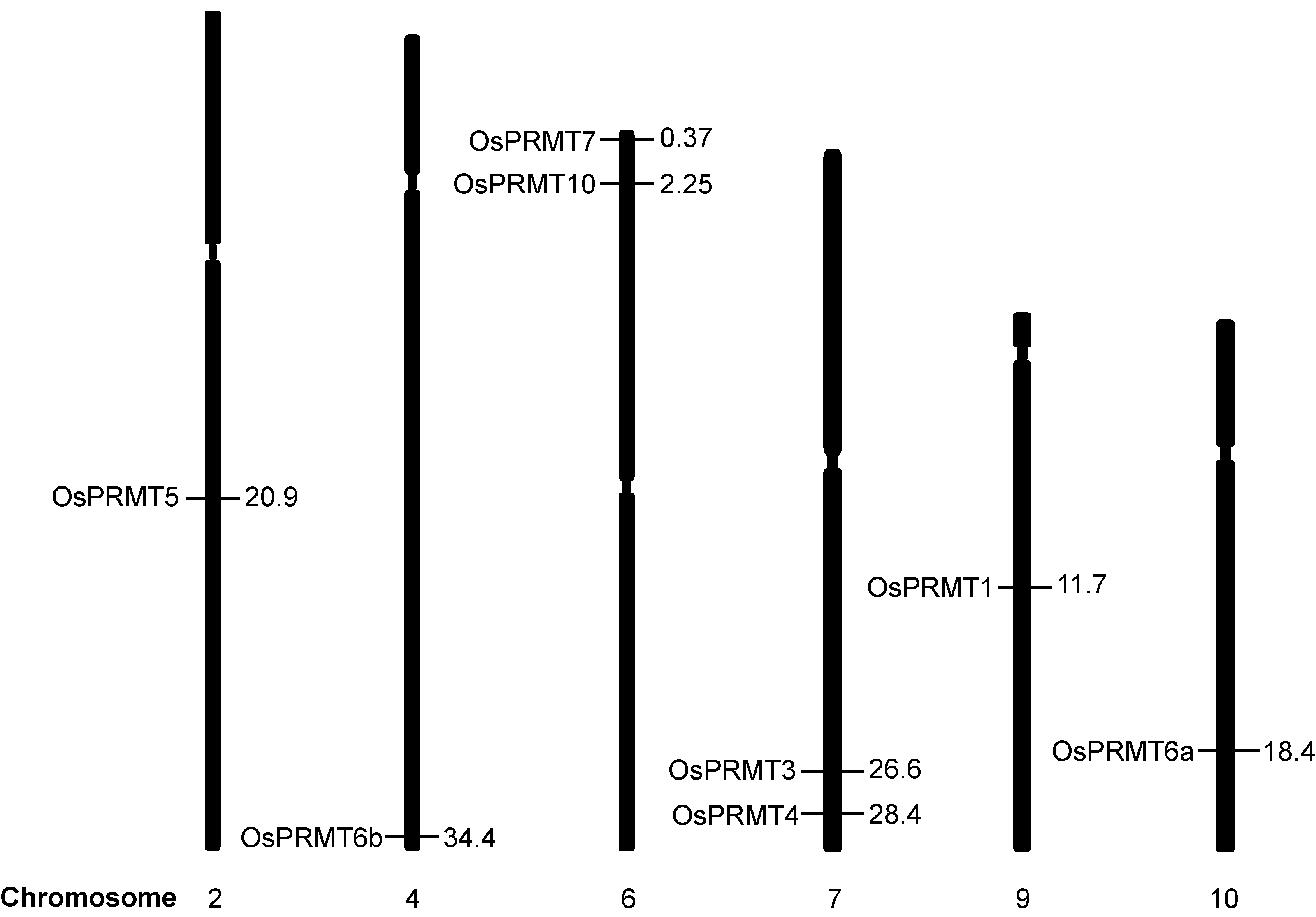

Supplement: Figure S1 — Chromosomal distribution of OsPRMTs. Chromosomal distribution of PRMT gene family member in Oryza sativa. Eight PRMT genes are distributed throughout the Oryza genome as single genes. Gene positions on the physical map of NCBI mapviewer are indicated in megabases for each gene. Six chromosomes on which eight PRMT genes localize are indicated by numbers (2, 4, 6, 7, 9, and10). (TIF) [file pone.0022664.s001.tif]

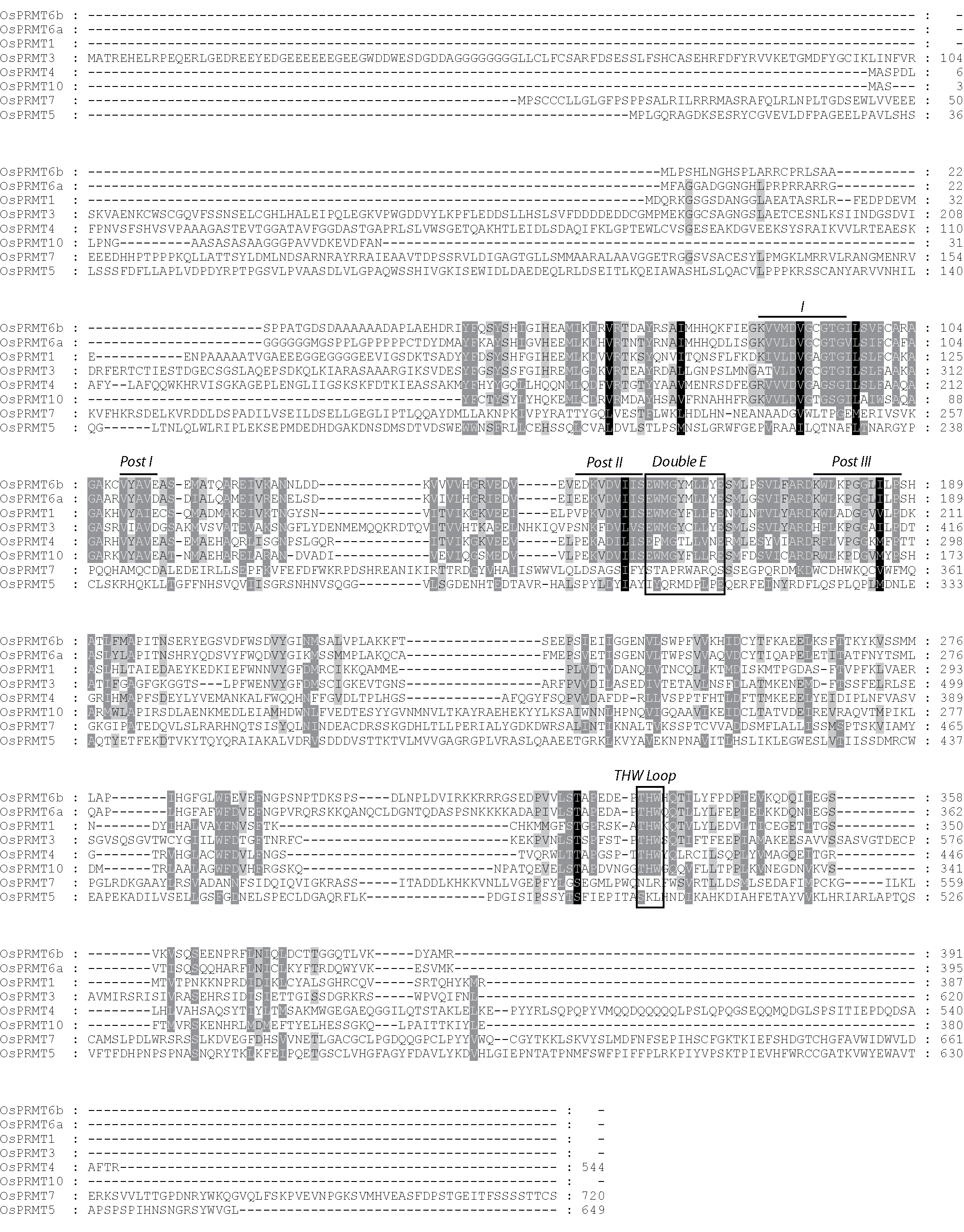

Supplement: Figure S2 — Amino acid sequence alignment of the PRMT gene family members in Oryza sativa . The TIGR/MSU identifiers for OsPRMT1, OsPRMT3, OsPRMT4, OsPRMT5, OsPRMT6a, OsPRMT6b, OsPRMT7 and OsPRMT10 are Os09g19560, Os07g44640, Os07g47500, Os02g04660, Os10g34740, Os04g58060, Os06g01640, and Os06g05090, respectively. Identical amino acids are boxed in black and similar amino acids are boxed in grey. Characteristic methyltransferase motifs, I, post-I, post-II, post-III are underlined, whereas Double E loop and THW loop are enclosed in boxes. (TIF) [file pone.0022664.s002.tif]

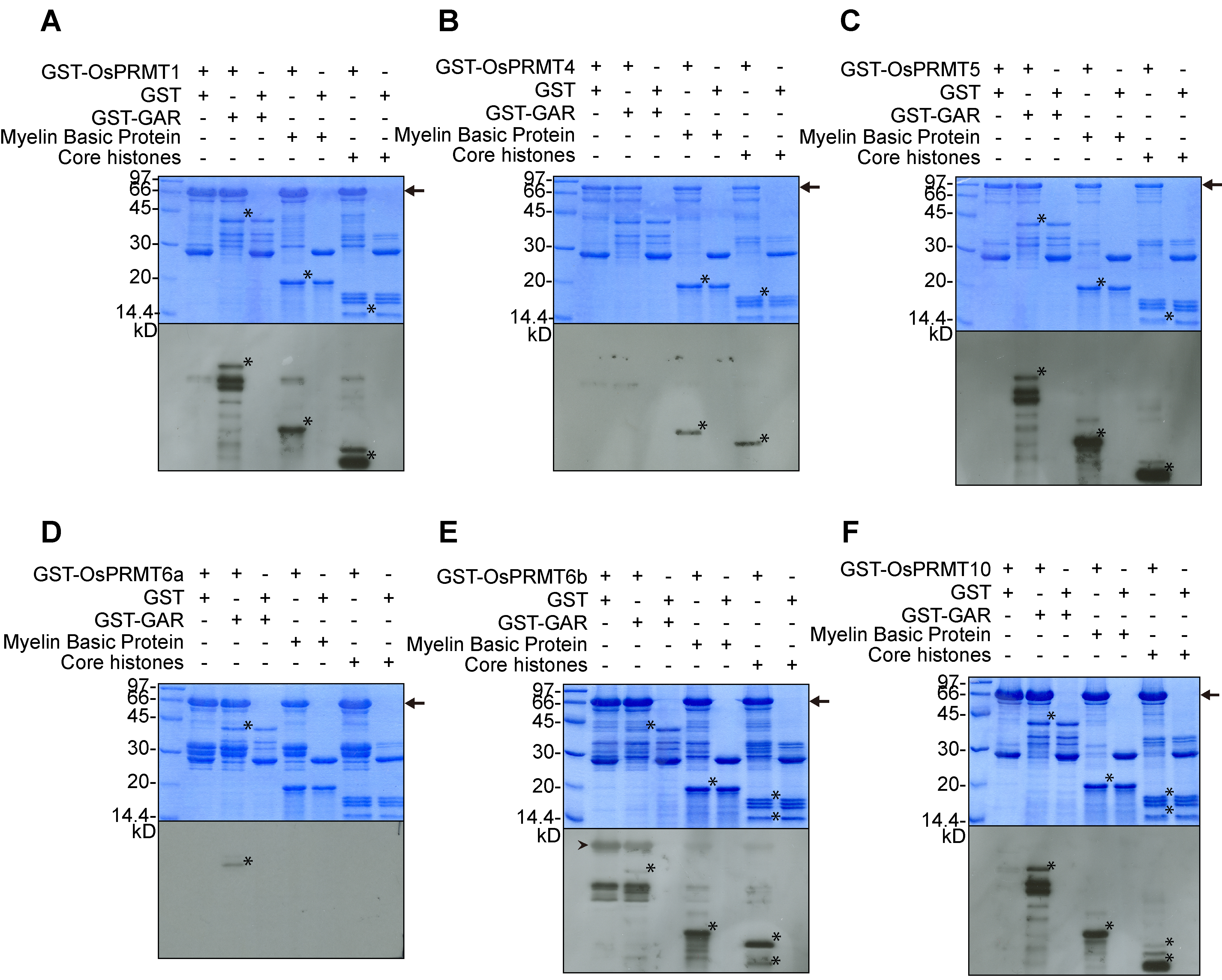

Supplement: Figure S3 — In vitro methyltransferase activity assay of the OsPRMTs (longer exposure time). S3A–S3F represent the in vitro enzyme activity assay result for the OsPRMT1, OsPRMT4, OsPRMT5, OsPRMT6a, OsPRMT6b and OsPRMT10, respectively. All the steps are essentially the same as in figure 3, however, the exposure time is four days. (TIF) [file pone.0022664.s003.tif]
