# Supplementary material for: Characterization of the PRMT Gene Family in Rice Reveals Conservation of Arginine Methylation
Source: PLoS One. 2011 Aug 11;6(8):e22664. doi: 10.1371/journal.pone.0022664 (PMC3154905; doi:10.1371/journal.pone.0022664)
Supplement: Table S1 — Table representing the identities/similarities of OsPRMTs to the Arabidopsis PRMTs at amino acids level. Amino acid sequences of the OsPRMTs and AtPRMTs were aligned in the bl2seq tool on the NCBI website to find the percent identity/similarity. (DOC) [file pone.0022664.s004.doc]

**Table S1.** Table representing the identities/similarities of OsPRMTs to the Arabidopsis PRMTs at amino acids level.

|  | **AtPRMT1a** | **AtPRMT1b** | **AtPRMT3** | **AtPRMT4a** | **AtPRMT4b** | **AtPRMT5** | **AtPRMT6** | **AtPRMT7** | **AtPRMT10** |
| --- | --- | --- | --- | --- | --- | --- | --- | --- | --- |
| **OsPRMT1** | 73/83 | 77/86 | 36/54 | 35/51 | 34/53 | 25/47 | 38/58 | 25/48 | 33/54 |
| **OsPRMT3** | 41/57 | 39/55 | 51/66 | 32/45 | 32/44 | 24/35 | 37/53 | 30/49 | 31/47 |
| **OsPRMT4** | 32/51 | 34/52 | 32/48 | 73/85 | 70/82 | 33/48 | 35/54 | 24/40 | 36/55 |
| **OsPRMT5** | 24/42 | 24/42 | 21/37 | 34/50 | 27/40 | 71/82 | 25/44 | 22/39 | 33/46 |
| **OsPRMT6a** | 42/60 | 41/59 | 39/57 | 32/51 | 32/52 | 24/46 | 64/82 | 27/50 | 34/54 |
| **OsPRMT6b** | 40/61 | 39/58 | 40/55 | 31/49 | 33/52 | 30/48 | 62/79 | 30/50 | 37/58 |
| **OsPRMT7** | 22/44 | 23/43 | 31/49 | 25/42 | 25/44 | 24/41 | 30/52 | 54/69 | 22/40 |
| **OsPRMT10** | 33/56 | 35/55 | 32/49 | 38/57 | 35/54 | 25/40 | 36/53 | 26/45 | 64/79 |
